# Extravascular leakage of dexrazoxane that occurred in a patient with diffuse large B-cell lymphoma: a case report

**DOI:** 10.1186/s40780-025-00446-1

**Published:** 2025-06-12

**Authors:** Nao Wakamiya, Masanori Suzuki, Tatsuya Isezaki, Ryohkan Funakoshi

**Affiliations:** 1 Postgraduate Center, Kameda Medical Center, 929 Higashi-cho, Kamogawa, Chiba 296-8602 Japan; 2 Faculty of Pharmaceutical Sciences, Teikyo Heisei University, Nakano-ku, Tokyo, Japan; 3 Department of Pharmacy, Kameda Medical Center, Kamogawa, Chiba Japan

**Keywords:** Dexrazoxane, Extravasation, Anthracycline antitumor agent, Case report

## Abstract

**Background:**

Dexrazoxane is used to treat extravascular leakage of anthracycline antitumor agents, but its own extravascular leakage and management remain underreported. This case aimed to highlight both doxorubicin and dexrazoxane leakage during treatment for diffuse large B-cell lymphoma.

**Case presentation:**

A 55-year-old man with diffuse large B-cell lymphoma developed doxorubicin leakage during rituximab, cyclophosphamide, doxorubicin hydrochloride, vincristine, and prednisolone (R-CHOP) therapy, which was treated with dexrazoxane. Subsequently, dexrazoxane leakage occurred, causing erythema and swelling. Topical clobetasol propionate was applied, leading to symptom resolution without necrosis. The patient successfully completed chemotherapy and achieved long-term remission.

**Conclusions:**

This case report is one of the first to document the management of dexrazoxane extravascular leakage using topical steroids, effectively preventing severe outcomes. The findings suggest that topical steroids may be a desirable treatment approach for dexrazoxane leakage. Prompt intervention and interdisciplinary care contributed to the favorable outcome. This case highlights the need for further research and guideline refinement to optimize the management of inflammatory extravascular leakage.

## Background

The incidence rate of malignant lymphoma is increasing every year [1]. Furthermore, diffuse large B-cell lymphoma (DLBCL) is the most common form of non-Hodgkin’s lymphoma in Japan [2], for which combination chemotherapy consisting of rituximab, cyclophosphamide, doxorubicin hydrochloride, vincristine, and prednisolone (R-CHOP) remains the first choice of treatment [3]; however, a serious complication associated with doxorubicin administration during R-CHOP is extravascular leakage.

The frequency of extravascular leakage due to necrotizing antitumor agents ranges from 0.1 to 6.5% [4], and that through central venous catheters is 0.4–0.7% [5]. Extravascular leakage of antitumor agents occurs at relatively low frequency, but even small amounts of extravascular leakage can damage the skin and surrounding tissues, which is characterized by redness, swelling, blistering, necrosis, ulceration, and severe pain. Early conservative treatment may be unsuccessful, resulting in skin necrosis and refractory skin ulcers requiring surgical intervention. Extravascular leakage causes a burden and distress to patients, such as reduced quality of life due to physical and psychological effects, increased risk of infection due to tissue damage, and a reduced number of vascular sites where antitumor agents can be administered due to vascular injury.

The most invasive drugs regarding the degree of skin tissue necrotic damage during extravascular leakage are necrosis-induced antitumor agents. Doxorubicin is classified as a DNA-binding necrosis-inducing antitumor drug that remains in the intracellular space and causes repeated damage, which may lead to long-term tissue damage and progressive ulcer formation [6–8]. Treatment with corticosteroids, bicarbonate, dimethyl sulfoxide, hyaluronidase, and α-tocopherol has been tried for extravascular leakage of antitumor agents [9, 10].

Dexrazoxane (Sabine® 500 mg for intravenous infusion) is a therapeutic agent used for extravascular leakage of anthracyclines such as doxorubicin [11]. Although it was initially developed as an antitumor agent [12], it is now considered to inhibit tissue damage caused by extravascular leakage. Dexrazoxane was approved in Japan in 2014 and is used in hospitals therein. It has been adopted in Kameda General Hospital since the same year and has been used in almost all cases of anthracycline leaks. Doxorubicin is also included in Pola-RCHP, the first-line therapy for DLBCL [13]. Consequently, the risk of extravasation requiring treatment with dexrazoxane, an agent used for anthracycline extravasation, still exists today. However, there are still no clear reports on the treatment of extravascular leakage of dexrazoxane.

We, therefore, aimed to present a case wherein dexrazoxane was administered for extravascular leakage of doxorubicin, an anthracycline antitumor agent, and the patient subsequently experienced extravascular leakage of dexrazoxane. This case has been reported with the approval of the Clinical Research Review Committee of Kameda General Hospital (Permission No.: Case 23 − 015).

## Case presentation

Patient: 55 years old, male.

Diagnosis on admission: Primary tonsillar DLBCL.

Stage: I.

Current medical history: In October 2017, a continuous mass was discovered from the right palatine tonsil and diagnosed as DLBCL. The hematology/oncology department opted to implement R-CHOP chemotherapy.

Extravascular leakage of doxorubicin occurred on Day 2 of the second course of R-CHOP, and dexrazoxane was administered. Pain and swelling at the puncture site were observed on Day 2 of dexrazoxane administration, and a diagnosis of extravascular leakage of dexrazoxane was made.

Medical history: None.

History of side effects, allergies: None.

Family history: No diabetes, heart disease, or cancer.

Activities of daily living: Independent.

On admission: height 167.8 cm, weight 63.5 kg, body surface area 1.72 m^2^, body mass index (BMI) 22.55 kg/m^2^.

### Clinical course of events

A pharyngeal mass was found in the right palatine tonsil at another hospital; a diagnosis of DLBCL was made after biopsy of the same area, and the patient was referred to our hematology/oncology department. A five-drug combination of R-CHOP was initiated as chemotherapy. Because constipation occurred after the first course, from the second course, vincristine was administered at a 50% dose, while all other drugs were administered at a 100% dose.

**Fig. 1:**
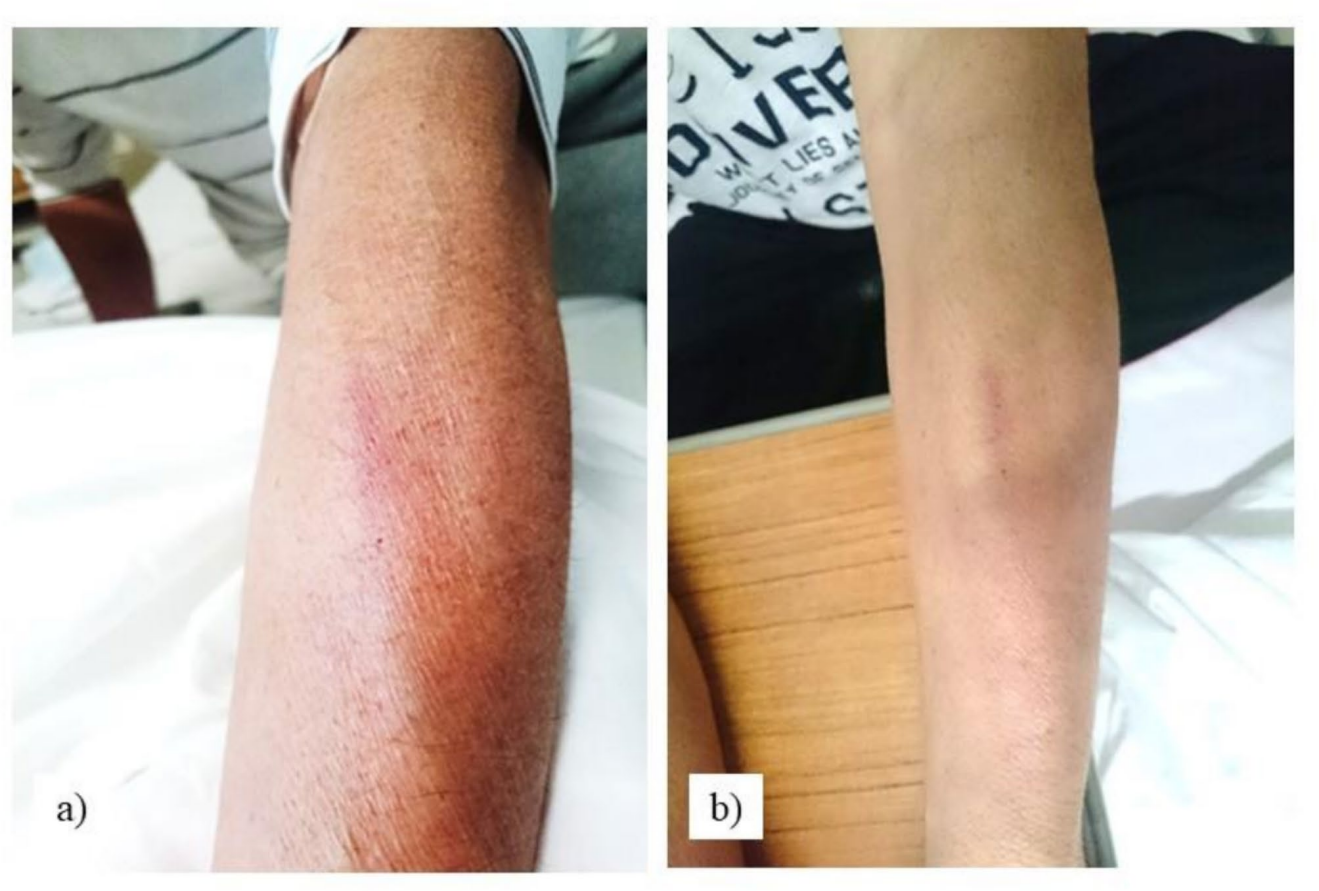
Doxorubicin leak sites and outcomes. (**a**) Doxorubicin leakage, Day 1. (**b**) Doxorubicin leakage, Day 17

On Day 1 of the second course, a 22-G indwelling needle was inserted into the left median forearm vein, and rituximab infusion (700 mg dissolved in 630 mL of 0.9% saline) was administered intravenously. Drug administration was completed without any adverse reactions. On Day 2, after confirming the reversal of blood loss, cyclophosphamide (1,300 mg dissolved in 500 mL of 0.9% saline) was administered intravenously. Thereafter, an infusion of doxorubicin hydrochloride (85 mg dissolved in 100 mL of 0.9% saline) was initiated. Twenty minutes after the start of dosing, an internal pressure of 100 mL of saline solution. and a supplemental solution of doxorubicin hydrochloride were applied, and the titration was determined to be faulty. The air needle was then punctured, but the infusion solution continued to fail to drip; therefore, the pharmacist in charge was consulted, and the air needle was punctured into the infusion solution body, but the solution did not drip. At this point, we also suspected obstruction on the venous side and attempted to draw reverse blood, but were unable to do so. There was no pain or swelling at this point, but erythema in the 1.5 cm x 2.5 cm range was noted along the vein from the incisional site to the central side (Fig. 1a).

A new line was established on the right side opposite the leak site, the remaining doxorubicin was resumed, followed by the administration of vincristine (1 mg in 100 mL of 0.9% saline), and the swollen area on the left arm was marked. Based on these circumstances, the pharmacist suspected extravascular leakage of doxorubicin and reported this to the hematologist, who requested the administration of dexrazoxane and concurrent dermatological consultation.

After examination, the physician suspected extravascular leakage of doxorubicin, and a 3-day infusion of dexrazoxane (1,700 mg dissolved in 100 mL distilled water followed by 500 mL lactated Ringer’s solution on days 1 and 2; 850 mg dissolved in 100 mL distilled water followed by 500 mL lactated Ringer’s solution) was initiated.

At the time of admission, the patient’s renal function was within the normal range (serum creatinine: 0.84 mg/dL; eGFR: 74.33 mL/min/1.73 m²), allowing for standard dosing of dexrazoxane. In addition to dexrazoxane administration, cooling and topical steroid application (clobetasol propionate ointment) were initiated twice daily. Because of the poor stability of dexrazoxane, an infusion pump was used, and dosing was initiated at 500 mL/h. Thirty minutes after the initiation of treatment, the patient complained of vascular pain. The administration rate was changed to 400 mL/h, and a warm towel was applied to the puncture site; however, there was no change in pain. Therefore, the administration rate was changed to 300 mL/h and continued thereafter.

The administration was completed within the period in which the stability of dexrazoxane was ensured. On Day 3 (Day 2 of dexrazoxane administration), 5 min after starting dexrazoxane administration at 500 mL/h, the patient complained of vascular pain. There was a 0.3 cm × 0.3-cm internal hemorrhage at the insertion site of the right forearm and pain. After replacement of the 22-G indwelling needle, administration was resumed at 300 mL/h of dexrazoxane using an infusion pump, after which the angialgia disappeared. There was no swelling or pain at the angialgia site.

On Day 4, there was no pain, and dexrazoxane administration was terminated, although a cord-like induration was observed in the right forearm wrist joint, the site where vasculopathy appeared the previous day. There was no complaint of pain in the left arm, but a central induration remained in the area where the indwelling needle was removed; some erythema and heat were also observed, which had become slightly more pronounced since the previous day.

**Fig. 2:**
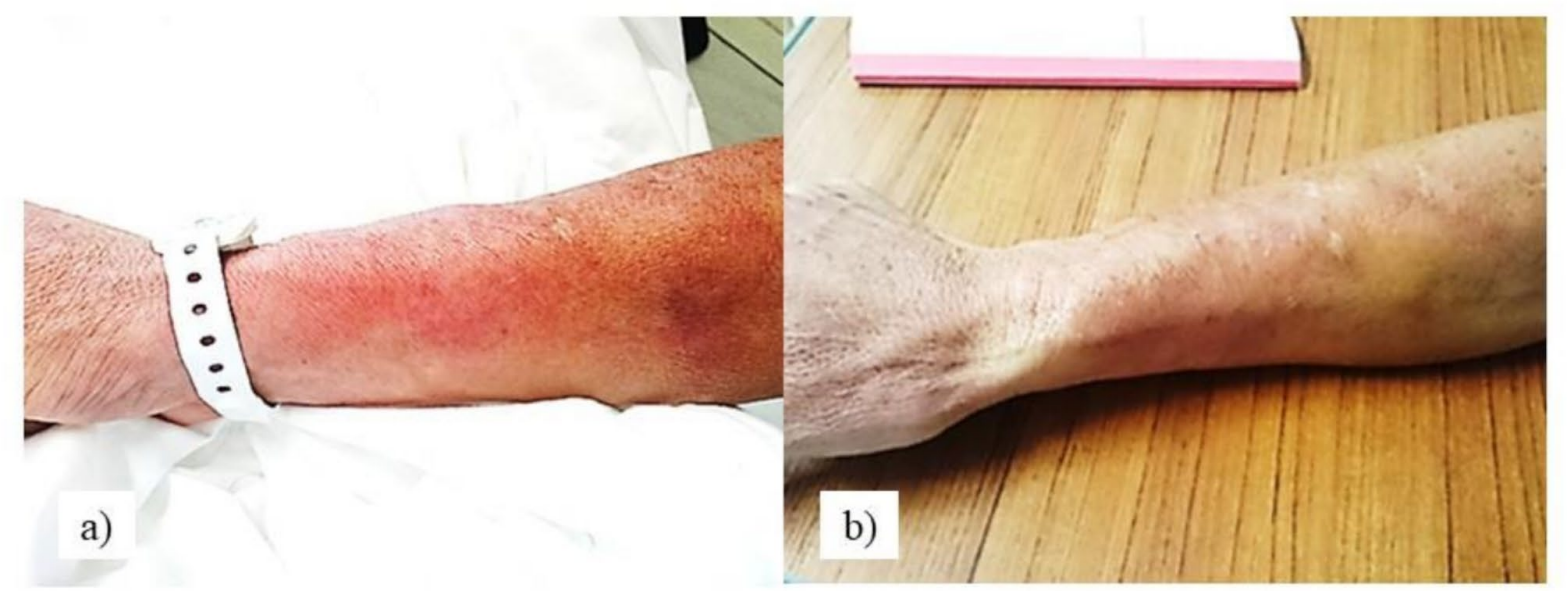
Dexrazoxane leak sites and outcomes. (**a**) Dexrazoxane leakage, Day 1. (**b**) Dexrazoxane leakage, Day 15

On Day 7, the patient complained of swelling and redness at the dexrazoxane administration site on the right forearm, with a numeric rating scale score of 5. A slightly bulging swelling (10 cm × 7 cm) with erythema and tenderness without induration was observed along the central vein from the dexrazoxane extraction site (Fig. 2a), and numeric rating scale level 3 pain persisted. Because phlebitis or inflammation associated with dexrazoxane leakage was suspected, the patient was referred to a hematologist. The patient was diagnosed with a dexrazoxane leak, and a dermatological follow-up was planned.

Information was collected regarding the risk of extravascular leakage due to dexrazoxane and the appropriate intervention. Injection site reactions occur at the site of dexrazoxane [14], and dexrazoxane itself is an inflammatory drug [15]. A clobetasol propionate ointment was prescribed to treat the affected area in accordance with the response to an inflammatory drug leak, and the patient began application to the affected area twice daily.

On Day 9, mild induration, swelling, and dark redness persisted on the right forearm, but the pain disappeared; however, the dermatologist examined the patient on Day 10, and the cord-like induration, which was palpable from the right forearm to the wrist on Day 8, was no longer detectable upon examination. Topical steroid application to the site of doxorubicin leak was discontinued, and topical dexrazoxane to the leak site was continued. On Day 11, erythema of the right forearm persisted, but the erythema and swelling tended to disappear, and the induration and pain disappeared. However, on Day 15, erythema tended to shrink, and pigmentation was observed, but there was no pain or ulceration.

On Day 17, the induration around the doxorubicin leak had disappeared (Fig. 1b), and no induration of the dexrazoxane leak was palpable (Fig. 2b); therefore, the patient was judged to be doing well, and the dermatologist completed the concurrent consultation. After the third course of R-CHOP, the patient was discharged without extravascular leakage. The patient did not develop any ulceration or serious skin lesions at the leakage site, and did not experience any serious adverse events associated with dexrazoxane, such as delayed myelosuppression or liver dysfunction. The patient was able to complete the rest of the courses of R-CHOP as an outpatient without any change in the scheduled chemotherapy.

**Fig. 3:**
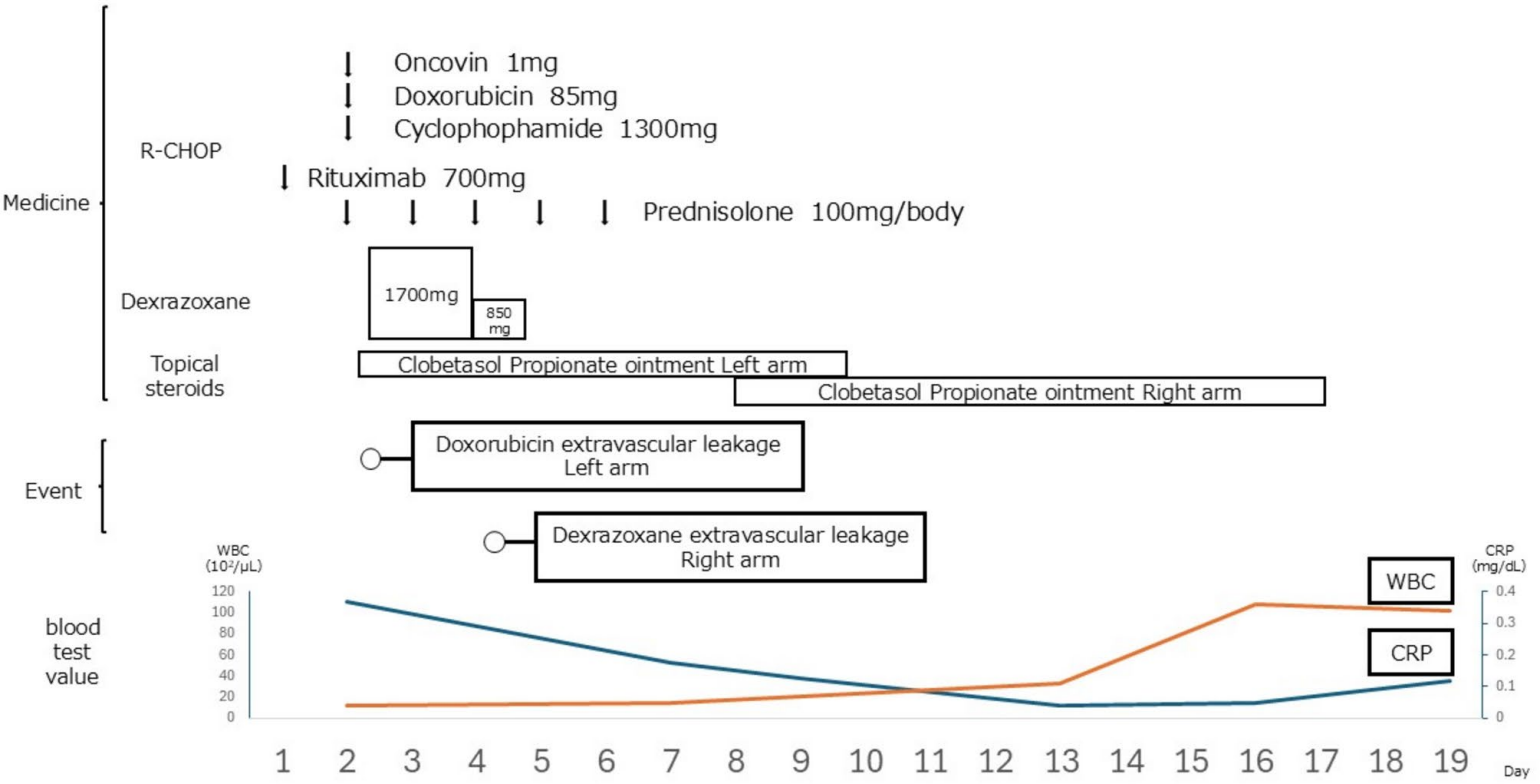
Progress. The course of the drugs administered, the events that occurred, and the blood laboratory values (white blood cells, C-reactive protein [CRP]) are represented)

The patient responded positively to the application of clobetasol propionate to the site of dexrazoxane leakage, saying that he no longer awoke at night with pain after applying the ointment and felt that it was working. Figure 3 shows the clinical course.

## Discussion and conclusions

In this case, doxorubicin, an anthracycline antitumor agent, leaked, and the patient was treated with dexrazoxane under medical supervision; however, extravascular leakage of dexrazoxane was observed. Dexrazoxane was initially developed as an antitumor agent [12] and has topoisomerase II inhibitory activity [16], which poses a risk of extravascular leakage. At the time of the extravascular leak in 2017, there were no general recommendations for dexrazoxane leaks; however, we acted based on the literature, classifying it as an inflammatory agent. The European Society for Medical Oncology [17] and the National Cancer Control Programme have prepared guidelines for the management of extravascular leaks, with no specific recommendations on how to respond depending on the degree of skin tissue necrosis. Although no specific response recommendations have been made, the guidelines prepared by the East Midlands Cancer Alliance recommend the use of topical steroids (weak steroids) in response to leaks of inflammatory antitumor agents. Additionally, the 2023 edition of the Guidelines for the Management of Chemotherapy Extravasation in Japan recommends the use of topical steroids [18].

We reviewed the literature for previous reports of extravascular leakage of dexrazoxane, like the present case, and summarized the reports that focused on the extravascular leakage of anthracyclines in Table 1 [19–26]. In most cases of anthracycline extravascular leakage since the launch of dexrazoxane, dexrazoxane has been administered. In about half of the cases, topical steroids were administered in combination with dexrazoxane. No necrotic tissue was observed in the dexrazoxane-treated cases. In cases where only dimethyl sulfoxide was administered, necrotic tissue was reported, and there were reports of procedures such as skin grafts and skin valves [19]. There are few reports on the outcome of the present disease, with the longest follow-up being up to 1 year and 6 months after treatment [24]. There have been no reports of extravascular dexrazoxane leakage in any patient. Case reports of common extravascular leaks have reported that the use of topical steroids prevents serious outcomes such as necrotic tissue; the strongest steroids were used in this case. In the latest edition of the Guidelines for the Management of Chemotherapy Extravasation [18], the inflammatory classification of dexrazoxane prepared in 2023 is not listed.

**Table 1:** Review of the literature regarding reports that focus on the extravascular leakage of anthracyclines

Case	Age	Sex	Cancer Type	Leaking agent	Leakage site	Dexrazoxane administration	Intravenous steroid	Topical steroid	Necrotic tissues	Treatment	Leackage of Dexrazoxane	Outcome of current disease	Reference
1-i	47	F	breast cancer	epirubicin	forearm	-	-	-	Yes	Skin graft	-	-	[19]
1-ii	66	F	breast cancer	epirubicin	Cubital fossa	-	-	-	Yes	Brachioradialis fap	-	-	
1-iii	49	F	breast cancer	epirubicin	forearm	-	-	-	Yes	Skin graft	-	-	
1-iv	78	F	breast cancer	epirubicin	Dorsum of the hand	-	-	-	Yes	Radial forearm fap	-	-	
1-v	45	F	breast cancer	epirubicin	Pectoral region	-	-	-	Yes	Latissimus dorsi fap	-	-	
1-vi	65	M	B-cell lymphoma	doxorubicin	forearm	-	-	-	Yes	Skin graft	-	-	
2	38	F	breast cancer	epirubicin	left forearm	-	Yes	Yes (details unknown)	Yes	Skin graft	-	-	[20]
3	70	F	malignant lymphoma	Mitoxantrone	left forearm	Yes	Yes	Clobetasol	None	-	-	-	[21]
4	-	-	esophageal adenocarcinoma	epirubicin	right forearm	Yes(cardioxa)	-	-	None	-	-	90 days of follow-up	[22]
5	42	F	breast cancer	doxorubicin	Infusion Port	Yes	-	-	None	-	-	Evaluation up to 6 months,postoperative outcomes are described.	[23]
6-i	50	F	follicular lymphoma	doxorubicin	right forearm	Yes	-	Betamethasone	None	-	-	Treatment completed, CR maintained (1 year and 4 months later)	[24]
6-ii	51	F	uterine cancer	doxorubicin	left forearm	Yes	-	-	None	-	-	Treatment completed, CR maintained (1 year and 6 months later)	
6-iii	51	F	uterine cancer	doxorubicin	right forearm	Yes	-	-	None	-	-	Treatment completed, CR maintained (1 year and 6 months later)	
7-i	76	M	Follicular lymphomas	doxorubicin	left forearm	Yes	-	Clobetasol	None	-	-	Treatment completed (8 courses)	[25]
7-ii	59	F	DLBCL	doxorubicin	left forearm	Yes	-	Clobetasol→Betamethasone、Acrinol	None	-	-	Treatment completed (8 courses)	
7-iii	64	M	Extensive small cell lung cancer	amrubicin	left forearm	Yes	-	Clobetasol	None	-	-	CV port implanted and treatment continued	
7-iv	78	M	invasive thymoma	amrubicin	left forearm	Yes	-	Clobetasol	None	-	-	-	
8	Unknown(30s)	F	AML	Daunorubicin/cytarabine	Left chest wall port	Yes	-	-	None	Dimethyl sulfoxideVancomycin	-	Death	[26]
9	55	M	DLBCL	doxorubicin	left forearm	Yes	-	Clobetasol	None	-	Yes	Treatment completed, CR maintained (6 years)	Our Case

In addition to the application of the ointment, the administration rate slowed as soon as vascular pain appeared, which was completed within the time required to ensure stability [11] after dexrazoxane preparation. Furthermore, we believe that the smooth exchange of information between nurses and pharmacists when extravascular leakage of dexrazoxane was detected and the daily sharing of symptom progress enabled the establishment of a system to promptly report to a hematologist–oncologist and dermatologist and request medical consultation when symptoms worsened after 3 days, which contributed to patient outcomes. The importance of establishing an in-hospital system for the administration of dexrazoxane for extravascular leakage of anthracyclines has been reported [23]. Our hospital has established an administration response flow for dexrazoxane administration, which may have had a small influence on its effectiveness. As for the response to the extravascular leak, prompt action was taken after the discovery of the leak, including confirmation of reverse bleeding, removal of the line, and consultation with a dermatologist, and the doxorubicin hydrochloride extravascular leak reduced without necrosis 6 days later. In addition, for dexrazoxane extravascular leakage, we applied steroid ointment as a topical treatment for inflammatory extravascular leakage, and the symptoms showed a tendency to lighten after 7 days. Therefore, we believe that our initial response was sufficient.

**Fig. 4:**
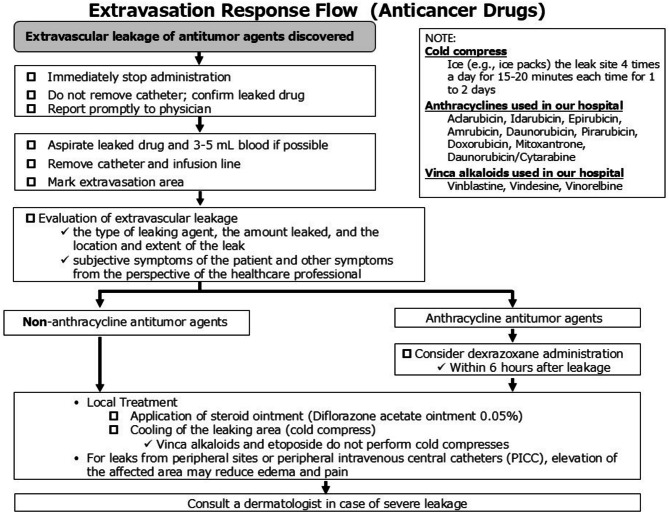
Extravascular leakage response flow. This is our hospital’s response flow in case of extravascular leakage of an anticancer drug

Various risk factors for extravascular leakage have been reported [17]; in this case, there were no clear factors, except that this was the second course of chemotherapy. One of the factors may have been the extravascular leakage of doxorubicin, which had been administered earlier, and the right forearm being secured at the radial cutaneous vein, which is on the opposite side, where it is a thin vessel susceptible to body movement. We believe that understanding the patient’s background with a focus on risk factors [17], establishing an initial response flow when an extravascular leak is detected, and improving the education system for hospital staff will impact patient outcomes (Fig. 4).

The site of extravascular leakage of doxorubicin hydrochloride improved after dexrazoxane administration. Local irritation, skin discoloration, and atrophy, which are recognized local side effects of topical steroids, did not occur [27]. The most important lesson learned from this experience is that in the event of a dexrazoxane leak, the application of topical steroids can prevent serious outcomes. Although there are reports that dexrazoxane falls under the category of inflammatory agents, there have also been reports of skin necrosis due to extravascular leakage of inflammatory antitumor agents [28], and since there is a possibility of serious outcomes, even in the case of leakage of inflammatory agents, topical application may be recommended as an inflammatory agent, as in the case of necrosis-inducing agents. A limitation of this case is that the R-CHOP regimen included systemic administration of high-dose prednisolone, and there are no cases of leakage of dexrazoxane alone in the absence of systemic administration of high-dose steroids. Based on the outcome of this case, it appears that localized steroid injection or prophylactic debridement is not necessary as an additional treatment; however, if painful inflammation develops, topical steroids or other agents may be considered. We addressed this based on a study that classified dexrazoxane as inflammatory [15], but no reason was provided for therein. Dexrazoxane itself is a topoisomerase II inhibitor, which may be a factor contributing to its cytotoxic properties. Cytotoxicity due to topoisomerase II inhibition has not been clarified in animal studies. Therefore, it is desirable to elucidate the mechanism of cytotoxicity of dexrazoxane through basic research.

Our patient completed treatment and achieved remission without regimen modification or reduction in the antitumor agent dosage after doxorubicin and dexrazoxane extravascular leakage. Six years after the extravascular leak episode, the patient was alive without recurrence. Although this was a short-term case of extravascular leakage, it is significant because the decision to continue chemotherapy can affect the long-term outcomes of the patient.

## Data Availability

No datasets were generated or analysed during the current study.

## Abbreviations

DLBCL: Diffuse large B-cell lymphoma
